# Modulating Complex Secondary Metabolism in *Streptomyces rimosus* by Targeted Genome Engineering

**DOI:** 10.17113/ftb.64.01.26.9441

**Published:** 2026-02-15

**Authors:** Martina Avbelj, Lucija Slemc, Alen Pšeničnik, Špela Zver, Anastasija Lazova, Kristina Mervič, Khan Mohammad Sarim, Maja Paš, Antonio Starčević, Martin Šala, Miha Tome, Dušica Vujaklija, Hrvoje Petković

**Affiliations:** 1 Food Science and Technology Department, Biotechnical Faculty, University of Ljubljana, 1000 Ljubljana, Slovenia; 2 Department of Analytical Chemistry, National Institute of Chemistry, Hajdrihova 19, 1000 Ljubljana, Slovenia; 3 Division for Physical Chemistry, Ruđer Bošković Institute, 10000 Zagreb, Croatia; 4 Faculty of Food Technology and Biotechnology, University of Zagreb, 10000 Zagreb, Croatia; 5 National Institute of Biology, Večna pot 111, 1000 Ljubljana, Slovenia

**Keywords:** biosynthetic gene cluster, oxytetracycline, rimocidin, gene regulation, genome reduction

## Abstract

**Research background:**

Numerous biosynthetic gene clusters (BGCs) encoding unknown structures have been discovered in the genomes of diverse microorganisms, representing a potentially rich source of novel natural products. However, most of the identified BGCs do not seem to be active, since we cannot detect any corresponding metabolites. Therefore, a better understanding of the regulation and biosynthesis of secondary metabolites encoded by these so-called ’silent‘ BGCs is of great importance.

**Experimental approach:**

We conducted a bioinformatic analysis of the *Streptomyces rimosus* ATCC 10970 strain, a producer of the antibiotic oxytetracycline, focusing on the expression of identified BGCs. We then reviewed experimentally identified compounds and putative structures predicted from genome data and similarity to known metabolites. We analysed available data on the regulation of two major metabolites – oxytetracycline and rimocidin, and experimentally evaluated the effect of the deletion of two oxytetracycline-competing pathways. Finally, we evaluated the effect of overexpressing BGC encoding the biosynthesis of the carotenoid isorenieratene, which cannot be detected in the culture of the native strain.

**Results and conclusions:**

We identified 48 BGCs in the genome of *Streptomyces rimosus* ATCC 10970. However, only about 15 structures were predicted or identified in the culture of this strain. Transcriptional analysis of identified BGCs demonstrated a very high variability in expression strength. Interestingly, around 30 % of BGCs were ’silent’. *In trans* overexpression of one such silent BGC, encoding the biosynthesis of the carotenoid isorenieratene resulted in strong production of this metabolite, suggesting that silent BGCs are likely still functional. We also demonstrated that BGCs encoding two major metabolites, oxytetracycline and rimocidin, both derived from malonyl-coenzyme A (malonyl-CoA), are not competitive pathways. Surprisingly, deletion of one silent BGC, also derived from malonyl-CoA, has a very strong effect on the biosynthesis of oxytetracycline.

**Novelty and scientific contribution:**

We observed that the expression strength of genes from BGCs identified in *Streptomyces rimosus* does not correspond to the experimental data obtained from the engineered strains, suggesting much more complex regulatory mechanisms than previously thought. Engineered *Streptomyces rimosus* host strains thus represent a very good model system to study the expression of ’silent’ BGCs.

## INTRODUCTION

The development of affordable and high-quality genome sequencing technologies has enabled a better understanding of the comprehensive secondary metabolism of *Streptomyces* species (*1*). A single *Streptomyces* species encodes 40–60 biosynthetic gene clusters (BGCs), and most of the secondary metabolite structures encoded by these BGCs remain uncharacterised. Therefore, they represent a rich source of novel metabolites with potential medical and industrial importance. For example, approx. 11 000 BGCs have been identified in the genomes of 830 actinomycetes (*2*). However, only a small proportion of these metabolites can be identified or detected at low concentrations (<1 mg/L) in liquid cultures of actinobacteria. This presents a challenge for the isolation of target compounds for biological activity studies and further preclinical evaluation.

Moreover, a large proportion of identified BGCs do not produce corresponding metabolites and are often referred to as ’silent BGCs’ (*3*). Most of these BGCs encoded in the genomes of various microorganisms are likely functional. However, they are highly regulated, so the probability of their expression under standard laboratory conditions is very low (*4*). Current state of the art for activating silent BGCs can be divided into two fundamentally different approaches: a) heterologous expression of all BGCs in *Actinomyces* chassis strains and b) induction of silent BGCs in the native host (*5*).

Recent studies suggest that *Streptomyces* genome reduction can also affect BGC expression. In the last decade, large-scale genome reduction approaches ranging from 100 kb to almost 1 Mb have been used to optimise microbial genomes and further develop simplified and versatile strains or microbial chassis for the production of valuable products (*6*). Even the deletion of a single BGC can influence secondary metabolite biosynthesis or related competing pathways (*7*).

Genome reduction is thought to lower the metabolic burden of the host strain and thus increase the titre of the target product. However, despite efforts of genome reduction in various *Streptomyces* species, no significant increases in the titres of target secondary metabolites have been achieved (*6*, *8*).

*Streptomyces rimosus*, the producer of the first broad-spectrum antibiotic oxytetracycline (OTC), is one of the best-studied industrial *Streptomyces* species (*9*, *10*). More importantly, *S. rimosus* is an excellent model system for conducting diverse studies in genome reduction and activation of silent BGCs. By applying the clustered regularly interspaced short palindromic repeats-associated protein 9/beta-glucuronidase (CRISPR-Cas9/GusA) system, which we developed in recent years (*11*), we introduced highly specific large deletions into the *S. rimosus* ATCC 10970 genome. We have observed that expression of the *cas9* gene in *S. rimosus* often causes selection pressure-related rearrangements of Cas9-containing plasmids, with frequent loss of the *cas9* gene. The advantage of the CRISPR-Cas9/GusA system we established in the *S. rimosus* system is that we can confirm expression of the *cas9* nuclease gene, as confirmed expression of the reporter gene *gusA* (which causes a blue-pigmented colony phenotype of transformants on agar plates) also ensures expression of the *cas9* gene due to the transcriptional fusion of the *cas9* and *gusA* genes.

We introduced two large deletions of 145 and 240 kb, which we positioned close to the *otc* BGC, located towards the end of the linear chromosome of *S. rimosus*. The size and location of the deletions in the *S. rimosus* chromosome were based on comparative whole genome studies on the native ATCC 10970 strain and high OTC-producing strains (*12*).

Remarkably, the introduction of the 145 kb deletion near *otc* BGC profoundly influenced OTC titres, reaching production titres of some industrial *S. rimosus* strains previously used for OTC production (*9*). Thus, in a single engineering step, we achieved an industrially relevant OTC titre in ATCC 10970, which is an unprecedented result (*12*). We observed that OTC BGC genes are overexpressed more than 50-fold in the engineered *S. rimosus* 10970 Δ145kb strain; however, we cannot yet explain the mechanism underlying this phenotype.

Here, we analysed all identified BGCs in ATCC 10970 in great detail using bioinformatics approaches and compared their expression profiles based on the transcriptome data presented by Pšeničnik *et al*. (*12*). We also analysed literature data related to regulatory elements and regulation of two major metabolites produced by ATCC 10970: polyene rimocidin (RIM) and OTC. Next, we selected the silent BGC 3, which encodes the biosynthesis of the carotenoid isorenieratene, and evaluated its *in trans* overexpression in ATCC 10970 Δ145kb. Finally, we precisely deleted selected BGCs to evaluate the effects of competing pathways.

## MATERIALS AND METHODS

### Strains and plasmids

The bacterial strains used in this study are listed in Table S1 (*12*-*15*). *Escherichia coli* DH10β was used for plasmid propagation, while *E. coli* ET12567/pUB307 served as the donor strain for intergeneric conjugation. The wild-type strain *S. rimosus* ATCC 10970 (NRRL 2234; WT5260), also known as strain R7, was used. In the engineered strain *S. rimosus* ATCC 10970 Δotc, the entire OTC BGC was deleted (*15*). In *S. rimosus* ATCC 10970 Δotc Δ145kb, in addition to the OTC BGC deletion, a 145 kb chromosomal region was removed (*12*). The plasmids used in this study are listed in Table S2 (*12*, *15*, *16*). The construction of the plasmids is described in detail by Pšeničnik *et al*. (*12*) and Slemc *et al*. (*17*).

#### Construction of the plasmid for the deletion of BGC 42

For the deletion of BGC 42, CRISPR-Cas9 was used (*11*). A 10 kb DNA fragment inside BGC 42, located between the WT5260_078570 (transcription regulator) and WT5260_078640 (polyketide synthase (PKS)) genes, was selected for the deletion experiment. The Geneious tool (*18*) was used to design guide RNAs (gRNAs)* in situ* with a protospacer adjacent motif (PAM) sequence of 5'-NGG-3'. For the knockout, two gRNAs (named 1a and 1b) located at the 5' and 3' ends of the target region for deletion were used. In addition to the gRNA sequences, homologous regions (≈1 kb) at the edge regions were also designed and cloned into the final vectors. The gene for resistance to thiostrepton was inserted into the pREP_P1_cas9 vector by amplifying the gene with the oligonucleotide primers tsr_Fw_ClaI (5'-AAAAATCGATGCTAGCTAGAGTCGACCTGC-3') and tsr_Rw_BspOI (5'-AAAAGCTAGCGGGTGCGCGTGATTGCCA-3'). The PCR product was purified from the gel, cut with the restriction enzymes *ClaI* and *BspoI*, and ligated into the pREP_P1_cas9 vector cut with the same enzymes. The resulting pREP_P1_cas9_tio vector was verified with restriction enzymes. The gRNA sequences (1a and 1b) and homologous regions were obtained in the pGH_GBG42_1a+1b+homology vector from DNA synthesis (ATG:Biosynthetics GmbH, Merzhausen, Germany). The entire sequence of the homologous regions and the gRNA region were excised from the pGH_GBG42_1a1b+homology vectors with the *XbaI* and *BstBI* enzymes and ligated into the pREP_P1_cas9_tio vector, linearized with the same enzymes. The resulting vector was named pREP_GBG42_1a1b and verified by restriction reaction after isolation from *E. coli* DH10β.

#### Construction of the integrative plasmid pAB04ErmE*EIBVYU containing the *crt* BGC 3 under the constitutive ermE* promoter

The plasmid pAB04ErmE*EIBVYU contains all genes for isorenieratene biosynthesis under the control of the strong constitutive promoter ermE*. The pAB04 vector was linearized with the restriction enzymes *XbaI* and *NdeI* and dephosphorylated with alkaline phosphatase. The isorenieratene BGC 3 was amplified by PCR in two parts, which contained the crtEIBV (EIBV_Fw TGTCGTACACTCACCCCGGAAACCTCCC; EIBV_Rw ACTCCAAGGAGGACCCCACAATGCGCCCTATCCGGGCA) and crtYU (YU_Fw GCTTGGGCTGCAGGTCGACTTCAGGCTCCCGCCACCCG; YU_Rw TCCGGGGTGAGTGTACGACACGGAGGTGGCC) genes, respectively. The oligonucleotide primers for the EIBV (CrtEIBV_Fw and CrtEIBV_Rw) and YU (CrtYU_Fw and CrtYU_Rw) parts were designed to introduce a 20–25 bp long homology at both ends of the fragments, which is necessary for the Gibson method of DNA fragment assembly. Genomic DNA isolated from ATCC 10970 using a bacterial genomic DNA isolation kit (Sigma Aldrich, Merck, St. Louis, MO, USA) was used as the template for the PCR reaction. The three fragments, EIBV, YU and pAB04, were then assembled using the Gibson DNA assembly kit (New England Biolabs, Ipswich, MA, USA).

### Cultivation conditions

*E. coli* strains were cultivated on solid or in liquid 2× yeast extract–tryptone medium (2TY) (*19*) at 37 °C. Transformed *E. coli* cells were grown in 2TY medium supplemented with appropriate antibiotics for 24–36 h, either in an incubator or on a shaker (Infors HT, Bottmingen, Switzerland) at 220 rpm. Intergeneric conjugation was conducted following a previously described protocol (*20*). *S. rimosus* exconjugants were selected on soya-mannitol (MS) agar plates supplemented with nalidixic acid (25 µg/mL) and thiostrepton (30 µg/mL). After initial selection, colonies were streaked onto fresh MS plates containing the appropriate antibiotic and incubated at 28 °C for 7 days. All *S. rimosus* strains used in this study (Table S1 (*12*-*15*)) were cultivated on MS agar to promote sporulation and in tryptone soy broth for genomic DNA isolation and plasmid removal by subcultivation. Fermentations for OTC production were carried out in 50-mL Falcon tubes containing 5 mL of medium. Seed cultures were initiated by transferring an agar plug from sporulated colonies into 5 mL of GOTC-V medium (in %: tryptone 5, glucose 1, calcium carbonate 0.1 and yeast extract 0.5), followed by incubation for 30 h at 28 °C with shaking at 220 rpm. Subsequently, *φ*(seed culture)=10 % were used to inoculate 5 mL of GOTC-P production medium, which was incubated for 5 days at 28 °C, 220 rpm and 60 % relative humidity (*21*). For carotenoid production, the same GOTC-V medium was used, while the production phase was conducted in liquid MS medium under the incubation conditions described above.

### Genomic DNA isolation

To isolate genomic DNA from *S. rimosus* ATCC 10970, *S. rimosus* M4018, and *S. rimosus* R6-500, a plug from sporulating colonies on MS agar was transferred into 5 mL of tryptone soy broth (in %: casein peptone 1.7, soy peptone 0.3, glucose 0.25, sodium chloride 0.5 and dipotassium hydrogen phosphate 0.25 (*20*)) and incubated at 28 °C with shaking at 220 rpm. Cells were harvested during the mid-exponential growth phase. Genomic DNA was extracted using the GeneElute Bacterial Genomic DNA Kit (Sigma-Aldrich, Merck) following the manufacturer’s protocol.

### Construction of the engineered strain

#### Deletion of BGC 42

BGC 42 in *S. rimosus* was deleted using a CRISPR-Cas9 system. The gRNAs targeting the cluster and homologous flanking sequences were cloned into plasmid pREP_P1_Cas9_tio by restriction cloning. *S. rimosus* transformants were selected on MS agar supplemented with thiostrepton (30 µg/mL) and theophylline (4 mM). The latter acts as an inducer of the theophylline-responsive riboswitch controlling Cas9 expression. Colonies were then restreaked onto MS agar with theophylline, and the presence of the desired deletion was confirmed by colony PCR. Positive clones were transferred to MS plates without antibiotics and, as a control, to MS plates with thiostrepton. Colonies that lost the plasmid did not grow under thiostrepton selection, consistent with the instability of the pIJ101 replicon present in pREP_P1_Cas9_tio.

### Identification and analysis of BGCs

BGCs in the genome of ATCC 10970 were identified using antibiotics and secondary metabolite analysis shell (antiSMASH) 6.0 (*22*). Each predicted cluster was then inspected manually with the analysis tools provided in AntiSMASH. The identified BGCs were compared with characterized clusters available in the minimum information about a biosynthetic gene cluster (MiBIG) database (*23*). Homologous genes in selected clusters were additionally examined using basic local alignment search tool (BLAST) searches (*24*).

### Quantification of OTC by HPLC

After *S. rimosus* strain cultivation, OTC production was determined as previously described by Pikl *et al*. (*15*). Briefly, 1 mL of production broth was acidified to pH=1.5–2, and samples were centrifuged at 18 000×*g* for 10 min (Megafuge 16R; Thermo Fisher Scientific, Osterode am Harz, Germany). The supernatant was filtered (Chromafil RC, 15 mm, pore size 0.45 µm; Macherey-Nagel, Düren, Germany) and analysed by HPLC (UltiMate 3000 system; Thermo Fisher Scientific, Waltham, MA, USA) equipped with a C18 column (150 mm×4.6 mm, 5 µm, 40 °C; Macherey-Nagel) and UV detection at 270 nm. The mobile phase consisted of solvent A (80 % water, 20 % methanol and 0.1 % formic acid) and solvent B (100 % methanol), applied in a 20 min gradient as follows: 0→8 min, 10 % B; 8→12 min, 10→90 % B; 12→15 min, 90 % B; 15→15.01 min, 90→10 % B; 15.01→20 min, 10 % B.

### Quantification of RIM by LC-MS

LC-MS analysis was carried out on UltiMate 3000 UHPLC system (Thermo Fisher Scientific) coupled with a triple quadrupole/linear ion trap mass spectrometer (4000 QTRAP LC-MS/MS System; Applied Biosystems/MDS Sciex, Ontario, Canada). Acetonitrile (Chromasolv LC-MS grade, Fluka, Buchs, Switzerland) and water purified on a Milli-Q system from Millipore (Bedford, MA, USA) were used for the preparation of mobile phases, and formic acid (FA) from Fluka was used as a modifier. An analytical HPLC column Kinetex XB-C18 100A (2.1 mm×100 mm, 2.6 µm particle size; Phenomenex, Torrance, CA, USA) was used with the flow rate of 0.3 mL/min (mobile phase A: water+0.1 % FA and B: acetonitrile+0.1 % FA, with the gradient of mobile phase B 0 min at 5 %, 15 min at 90 %, 18 min at 90 %, 18.1 min at 5 % and 23 min at 5 %). Injection volume and column temperature were 10 µL and 30 °C, respectively. High-resolution mass spectrometry (HMRS) measurements were performed with a hybrid quadrupole orthogonal acceleration time-of-flight mass spectrometer (QTOF Premier; Waters, Milford, MA, USA).

### Statistical analysis

Statistical analysis of OTC production as quantified by HPLC was performed using GraphPad Prism v. 10.1.2 (*25*). The experimental data collected in this study were processed with Dunnett’s T3 test, assuming unequal variances (*N*=9). Corresponding p-values are reported in figure legends.

## RESULTS AND DISCUSSION

### Identified BGCs in ATCC 10970

OTC-producing *Streptomyces rimosus* strains generally have a chromosome of about 9 Mb with a specific architecture: a conserved, highly syntenic central core of the genome flanked by dynamic and variable chromosomal arms (*26*). The genome of ATCC 10970, the progenitor of industrial strains with high OTC titre, has been sequenced intensively by different research groups (*27*-*29*) and consists of one linear chromosome of about 9 Mb and one linear plasmid of approx. 300 kb (*17*).

**Fig. 1:**
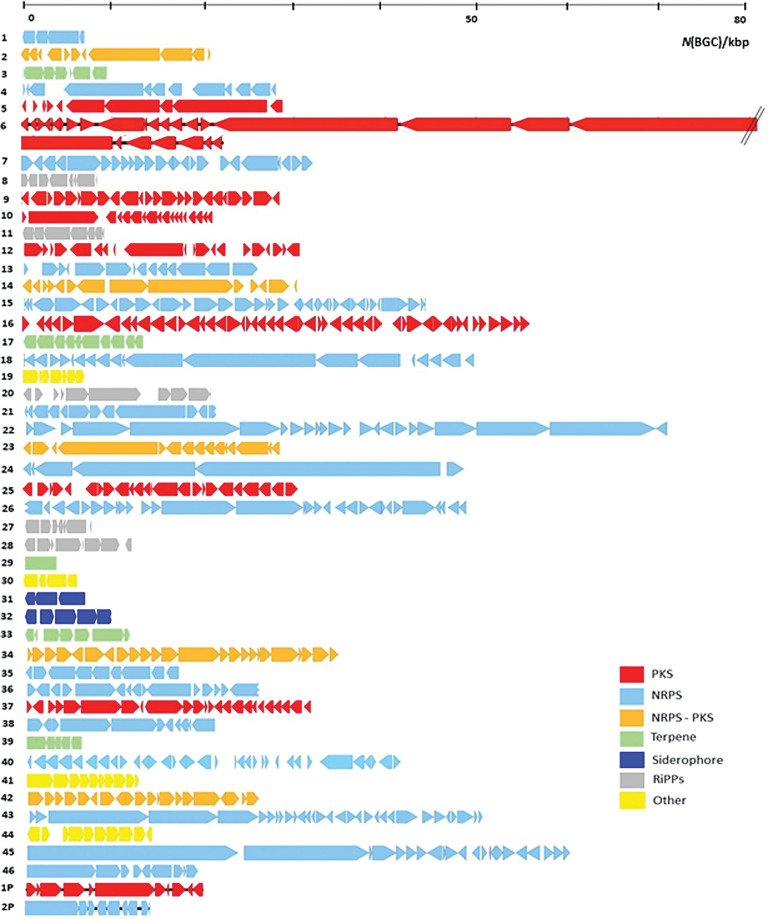
Putative biosynthetic gene clusters (BGCs) identified by the antiSMASH 6.0 tool (*22*) in the ATCC 10970 chromosome. The size of BGCs is expressed in kilobase pairs (kbp). The BGCs are colour-coded according to the type of biosynthetic enzyme complex. PKS=polyketide synthase, NRPS=non-ribosomal peptide synthetase, RiPPs=ribosomally synthesized and post-translationally modified peptides

Previously, a high-quality genome sequence (GCF_006229535.1) was obtained from ATCC 10970 (*17*). Here, we used the antiSMASH 6.0 tool (*22*) to identify potential BGCs in the entire genome of ATCC 10970. We identified 46 BGCs on the chromosome, and two additional BGCs on the giant linear plasmid of about 300 kb (Table S3). The largest group of 16 BGCs belongs to non-ribosomal-peptide synthetase (NRPS) enzyme complexes. The remaining putative BGCs identified belong to the following groups: PKS type I (*N*=5), PKS type II (*N*=1), BGCs encoding combined NRPS-PKS complexes (*N*=6), BGCs encoding for the biosynthesis of terpenoids (*N*=5), BGCs encoding ribosomally synthesized and post-translationally modified peptides (RiPPs) (*N*=5), BGCs encoding siderophore biosynthesis (*N*=3), and BGCs encoding other enzymatic groups (*N*=6) (Table S3 and Fig. 1). The identified putative BGCs represent around 14 % of the entire genome of ATCC 10970 (*17*).

**Fig. 2:**
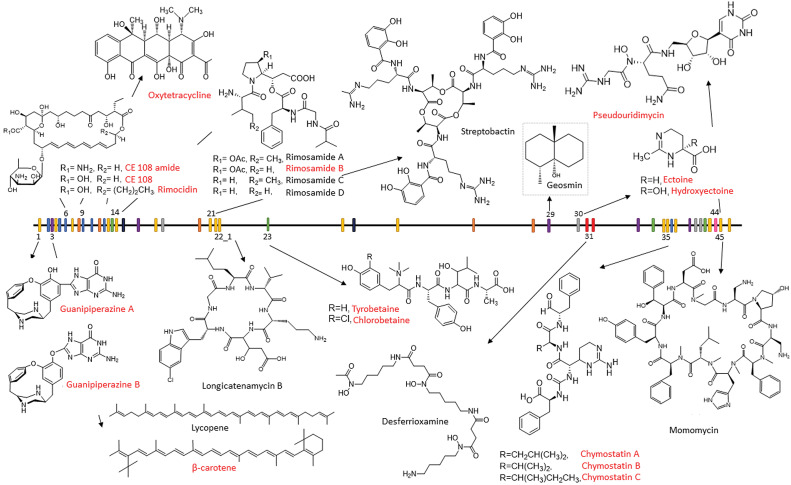
Secondary metabolites identified in ATCC 10970 to date. The distribution of biosynthetic gene clusters on the genome of ATCC 10970 and the structures of the corresponding secondary metabolites are shown. Secondary metabolites detected in ATCC 10970 cultures in this study are marked in red. Other structures, marked in black, have been described previously in the literature

Our results show that BGCs encoded by ATCC 10970 can be classified into three categories: (*i*) BGCs whose corresponding metabolites were identified experimentally in *S. rimosus* cultures, (*ii*) BGCs showing very high sequence similarity to known clusters previously identified in other *Actinomyces* species, and (*iii*) putative BGCs displaying minimal or no homology to any known BGC-encoding metabolite (Table S3 and Fig. 2). The identification and detailed description of BGCs encoded by ATCC 10970 are shown in the Supplementary material.

### Analysis of secondary metabolites in OTC-producing S. rimosus cultures

When using standard laboratory media for OTC production (*17*), the major products found in ATCC 10970 cultures were OTC and antifungal polyene rimocidin (RIM) (encoded by BGC 9 and BGC 6, respectively), both produced at approx. 100 mg/L. In our recent study (*12*), we confirmed that ATCC 10970 produces not only RIM, but also two RIM analogues, CE-108 and its corresponding amide form, which were previously reported only in *Streptomyces diastaticus* var. 108 (*17*, *30*).

In our recent study (*17*) (Table S3 and Fig. 1), we confirmed that ATCC 10970 produces secondary metabolites belonging to the thiopeptide-like compound group of tyrobetaines encoded by BGC 23 (*12*), rimosamides encoded by BGC 14 (*31*) and the nucleoside antibiotic pseudouridimycin encoded by BGC 44 (*32*). Specifically, we detected tyrobetaine, chlorotyrobetaine, tyrobetaine-2 and dichlorotyrobetaine encoded by BGC 23, rimosamide B (but not variants A or C) encoded by BGC 14, and pseudouridimycin encoded by BGC 44. These findings indicate that BGCs 14, 23 and 44 are expressed under laboratory conditions used in our study. Interestingly, however, BGC 14 expression was not detected in our transcriptome data (see subsection Regulation of secondary metabolism in *S. rimosus*).

*S. rimosus* NRRL B-8076 was previously reported to produce chymostatin B (*33*), and our results show that ATCC 10970 synthesizes chymostatins A, B and C, encoded by BGC 35 (*17*) (Table S3 and Fig. 1). We also confirmed the production of ectoine and hydroxyectoine (BGC 30 (*17*)), two metabolites commonly associated with osmoprotection and thermotolerance in streptomycetes (*34*). In addition, one study reported the production of streptobactin (BGC 21), longicatenamycin B (BGC 22), deferoxamine (BGC 31) and momomycin (BGC 45) in ATCC 10970 ((*35*); Table S3 and Fig. 1). By contrast, under our cultivation conditions, we only detected weak expression of BGC 45.

We also identified longicatenamycin B (BGC 22) in ATCC 10970 with a 145 kb deletion (*12*). Furthermore, we confirmed the production of guanipiperazine A and B (BGC 1) by ATCC 10970 (*17*). Following their isolation and structural elucidation, we identified the corresponding BGC 1, which shares homology with the *gup* BGC responsible for guanipiperazine A and B biosynthesis in *Streptomyces chrestomyceticus* (*36*). Importantly, our findings represent the first evidence of *gup* BGC expression in a native producer organism, as homologous clusters remain silent in other *Streptomyces* species (*36*).

To conclude, only 12 of its structures can be associated with their corresponding BGCs (out of 48 secondary metabolite BGCs), which is less than 30 % of the putative metabolites encoded in the genome of this *Streptomyces* species.

### In trans overexpression of BGC 3 encoding isorenieratene biosynthesis

Given the high sequence similarity of BGC 3 to the known isorenieratene BGCs, isorenieratene is presumed to be the primary product of BGC 3, while β-carotene and lycopene are intermediates in the biosynthesis of isorenieratene (*37*, *38*).

We selected BGC 3, which putatively encodes isorenieratene, for *in trans* expression of a chromosomally integrated copy because orange-to-red carotenoids represent a very simple reporter system. Interestingly, in our laboratory, red pigmentation was never observed in the various cultivation media used for OTC production with ATCC 10970 (*17*). As expected, the detected BGC 3 expression was very low (see subsection Regulation of secondary metabolism in *S. rimosus*).

**Fig. 3:**
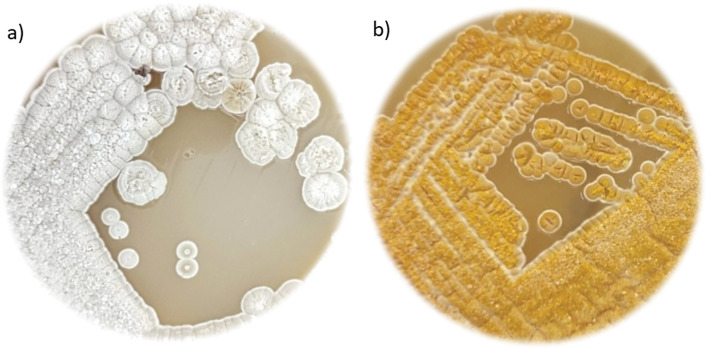
*Streptomyces rimosus* ATCC 10970 Δotc Δ145kb colonies: a) without and b) with the chromosomally integrated plasmid pAB04 harbouring a version of the engineered isorenieratene BGC 3 expressed under the strong constitutive promoter ermE*. BGC=biosynthetic gene cluster

To evaluate the functionality of the BGC 3 enzyme complex that encodes isorenieratene biosynthesis, we constructed the ATCC 10970 Δotc Δ145kb strain with an *in trans* chromosomally integrated plasmid harbouring an engineered version of isorenieratene BGC 3, expressed under the strong constitutive promoter ermE*. Intensely pigmented colonies were easily observed, indicating carotenoid production (Fig. 3). We used the integrative plasmid pAB04 containing ØC31-based integrase (*15*). The plasmid integration site is located internally in the chromosome of *S. rimosus*, as described by Carillo Rincón *et al*. (*16*). Construction of the isorenieratene BGC 3 on the integrative plasmid pAB04 is described in the Materials and Methods section. Production of the carotenoid isorenieratene in the engineered strain was further confirmed by high-resolution mass spectrometry (for details, see Supplementary Data S1). This experiment demonstrates that isorenieratene BGC 3 is functional but not expressed sufficiently to enable identification of carotenoids in *S. rimosus* in the cultivation media favouring OTC production (*21*).

### The effects of competing pathways on OTC biosynthesis: deletion of selected BGC-encoded pathways

Generally, the inactivation or deletion of BGCs that potentially compete for substrates (building blocks) could be a valid strategy for the construction of engineered strains that produce new metabolites encoded by the silent BGCs. As shown in the literature, regulatory elements, including BGC-specific and general (pleiotropic) regulators, can influence the expression of silent BGCs, including transcriptional regulators. For example, the following regulatory proteins have been identified: LysR-type transcriptional regulators, *Streptomyces* antibiotic regulatory proteins, tetracycline repressor proteins, and arabinose operon regulatory proteins (*39*).

### Inactivation of RIM BGC 6 and combined PKS/NRPS BGC 42 in ATCC 10970

RIM and OTC are major metabolites present in ATCC 10970 cultures and can be considered competing pathways, as both BGCs encode polyketide synthase enzyme complexes, whose main substrate (building block) is malonyl-CoA. However, in the study by Pšeničnik *et al*. (*12*), precise in-frame inactivation of the *rim* PKS BGC 6 loading domain did not affect OTC titre. Interestingly, the result of this experiment, where production of RIM was completely abolished, suggests that malonyl-CoA substrate supply is not a bottleneck in OTC biosynthesis. As such, RIM BGC 6 and OTC BGC 9 do not seem to be competing pathways under the applied cultivation conditions. Additionally, this result suggests that no significant inter-regulation exists between these two BGCs.

**Fig. 4:**
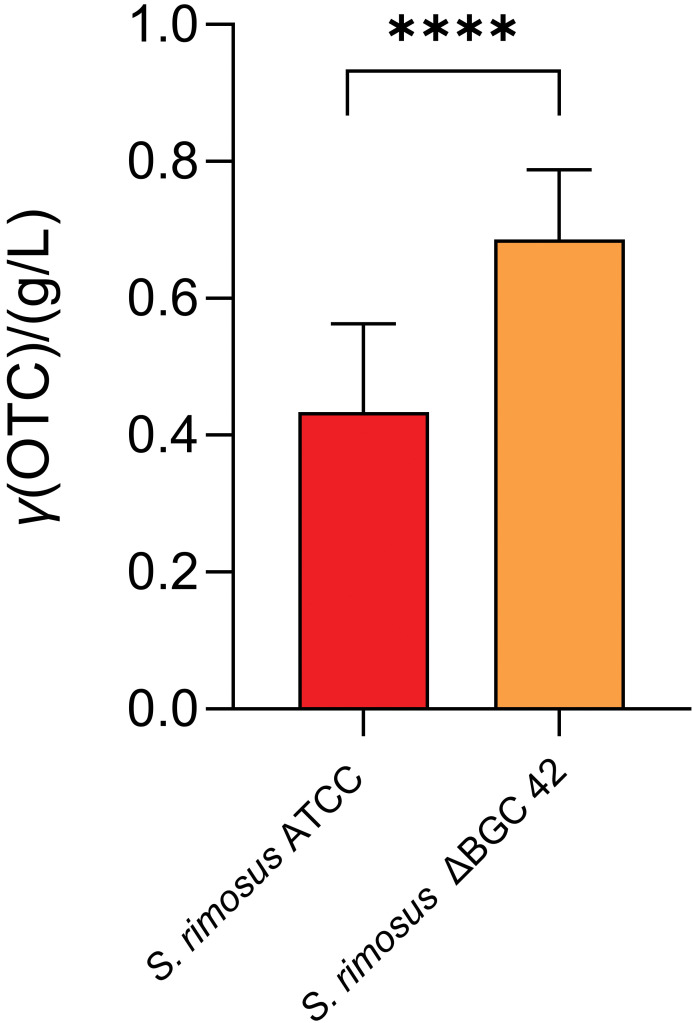
Deletion of biosynthetic gene cluster (BGC) 42 increases the oxytetracycline (OTC) titre in ATCC 10970. BGC 42 encodes combined PKS type I–NRPS enzyme complexes. PKS=polyketide synthase, NRPS=non-ribosomal peptide synthetase, RiPPs=ribosomally synthesized and post-translationally modified peptides. **** indicates statistical significance (p<0.0001)

We also evaluated the potential effects of competing pathways on OTC biosynthesis in ATCC 10970 and identified a pathway belonging to combined PKS type I–NRPS enzyme complexes, encoded by BGC 42 (Table S3). Our bioinformatics analysis and data from the literature suggest that the PKS type I portion of BGC 42 selects malonyl-CoA as a substrate (extender units). Thus, BGC 42 could potentially represent a competing pathway for *otc* BGC 9. Interestingly, compared to *otc* BGC 9, BGC 42 is located on the opposite side of the linear chromosome of ATCC 10970 (Table S3 and Fig. 2). Interestingly, our transcriptome studies show that this BGC is entirely silent, as no expression of BGC 42 could be observed (see subsection Regulation of secondary metabolism in *S. rimosus*). We precisely deleted BGC 42 in ATCC 10970 (as described in the Materials and methods and Supplementary material), which increased OTC titres by more than 70 % (Fig. 4). The increase in OTC titre in the strain with deleted BGC 42 is highly significant, which raises the question of how a transcriptionally silent BGC, whose corresponding metabolite has not been identified, can influence the biosynthesis of OTC.

As mentioned earlier, RIM is one of the major metabolites in ATCC 10970, where *rim* BGC 6 expression is significantly higher than *otc* BGC 9 expression. It is thus surprising that the inactivation of *rim* BGC 6 did not affect OTC biosynthesis. Conversely, although completely silent, inactivation of silent BGC 42, located on the other side of the linear chromosome (far from *otc* BGC 9), significantly increased OTC titre (Fig. 4).

### Regulation of the biosynthesis of OTC and RIM in ATCC 10970

OTC and RIM biosynthetically belong to the class of polyketide natural products and are major metabolites in *S. rimosus* (*9*, *12*). Therefore, it is possible that the corresponding BGCs that encode their biosynthesis are also strongly expressed. To evaluate the expression of RIM and OTC, as well as the other 46 BGCs identified in the genome of ATCC 10970, we conducted whole transcriptome analysis, as described by Slemc *et al*. (*17*) and Pšeničnik *et al*. (*12*).

**Fig. 5:**
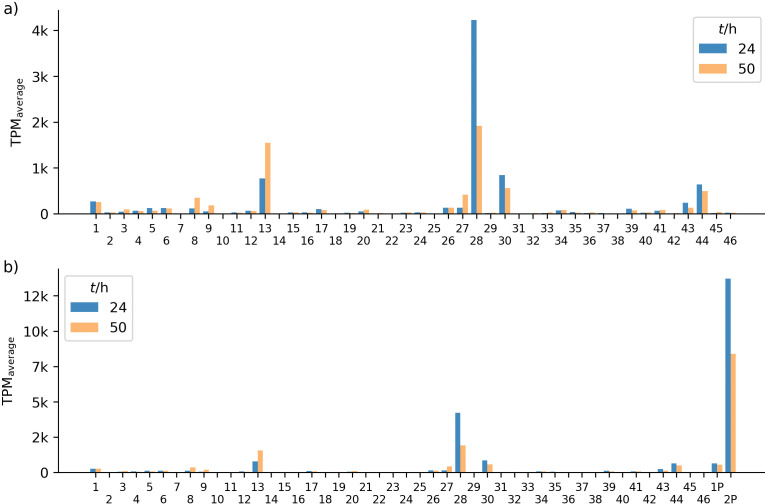
Comparative analysis of the expression of all identified biosynthetic gene clusters (BGCs) in ATCC 10970 cultures. Values are presented as average transcripts per million (TPM) after 24 and 50 h of incubation in laboratory production medium GOTC (*21*). The expression of BGCs located: a) solely on the chromosome, and b) on the chromosome and linear plasmid (labelled 1P and 2P). The x-axes show the BGC location on the chromosome. GOTC=laboratory medium for OTC production

Transcriptome data analysis (*12*) shows that the expression of 48 BGCs varies significantly, with transcripts per million (TPM) values ranging from close to zero to over 13 000. As expected, the average TPM value of a BGC does not correspond to the concentration of the respective secondary metabolite produced by ATCC 10970 (Fig. 5). For example, in ATCC 10970 cultures, the major metabolites OTC and RIM reach titres of around 100 mg/L, while the expression of the corresponding *otc* and *rim* BGCs is rather moderate, with TPM values of about 100 and 400, respectively. Notably, the TPM values of these two BGCs increase significantly after 50 h compared to 24 h, which is consistent with the observation that OTC biosynthesis is undetectable after 24 h but intense after 50 h. This pattern of higher TPM values after 50 h than after 24 h was also observed for BGCs 8, 13, 26 and 27 (Fig. 5). However, the products of these BGCs have not been identified (*12*).

Our transcriptome analysis showed that BGC 2P, which is located on the 300 kb linear plasmid and encodes putative NRPS, exhibits the strongest expression among all BGCs (Fig. 5b and Table S3). The highest TPM value of all BGCs located on the linear chromosome of ATCC 10970 is around 4000 (BGC 28; Fig. 5), a value much lower than that of BGC 2P (almost 14 000), located on the linear plasmid. A previous study by Slemc *et al*. (*17*), who applied pulsed-field gel electrophoresis, suggested multiple copies of the linear plasmid in a single cell, which could potentially contribute to the high TPM value of BGC 2P.

BGCs with average TPM values below 10 are considered weakly expressed (*40*). According to this criterion, 10 out of the 46 BGCs on the chromosome showed weak expression. Moderate expression with average TPM values of 10–100 was observed in roughly half of the BGCs at 24 h (23 BGCs) and in more than half (27 BGCs) at 50 h (Fig. 5).

It is also important to acknowledge potential limitations of the analytical methods used in our work (*12*, *17*). However, it is reasonable to expect that metabolites produced at titre of over 10 mg/L would be detected by LC/MS, as well as by other research groups that have been investigating ATCC 10970 for decades.

### Regulation of secondary metabolism in S. rimosus

#### Regulatory elements identified in OTC BGC and their function

The genome sequence of ATCC 10970 revealed that, like other streptomycetes (*39*), this strain encodes numerous genes predicted to play regulatory roles in various cellular processes. Over 10 % of the genome consists of genes involved in regulation. We recently reported dynamic changes in the synthesis and post-translational modification of numerous regulatory proteins, highlighting their prominent role in complex and dynamic signalling networks linked to growth arrest, metabolic switching, antibiotic production, and bacterial responses to nutrient deprivation and other stressors in this strain (*41*). However, to date, relatively few regulatory genes in *S. rimosus* have been studied in detail. The following section describes the experimentally characterized regulatory genes located within the OTC cluster (*10*, *42*, *43*).

**Fig. S1:**
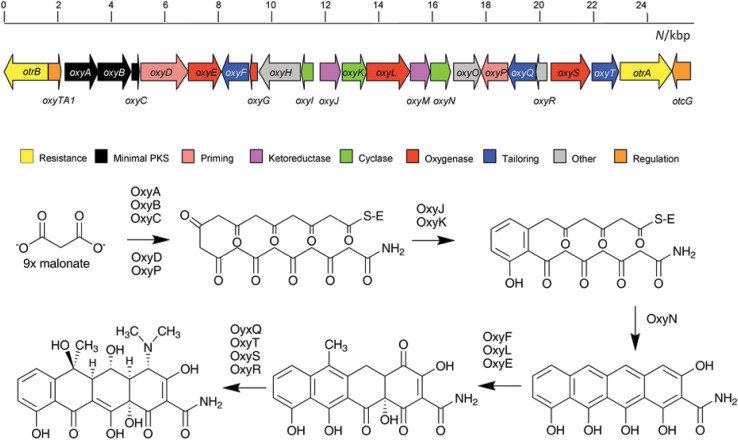
Proposed biosynthetic pathway and genes involved in oxytetracycline (OTC) biosynthesis. PKS**=**polyketide synthase

Yin *et al*. (*42*) identified the regulatory protein SARP (*Streptomyces* antibiotic regulatory protein), which acts as a transcription activator, OtcR, whose gene is located immediately adjacent to the resistance gene *otrB* (Fig. S1). OtcR acts as a positive pathway-specific activator of OTC biosynthesis and increases OTC production when overexpressed at the appropriate level (*42*).

The second regulatory element, OtcG, was identified by Lešnik *et al*. (*43*). OtcG belongs to the LAL (large ATP-binding regulator of LuxR) family of transcriptional regulators and is located on the other side of the *otc* BGC, near the *otrA* resistance gene (Fig. S1). OtcG plays a conditionally positive role in OTC biosynthesis, as inactivation of the *otcG* gene in the industrial strain *S. rimosus* 4018 (progenitor of ATCC 10970) reduces OTC production by >40 %. However, overexpression of *otcG*, by introducing a second copy of the gene under the strong constitutive promoter ermE*, did not significantly alter OTC production (*43*).

**Fig. 6:**
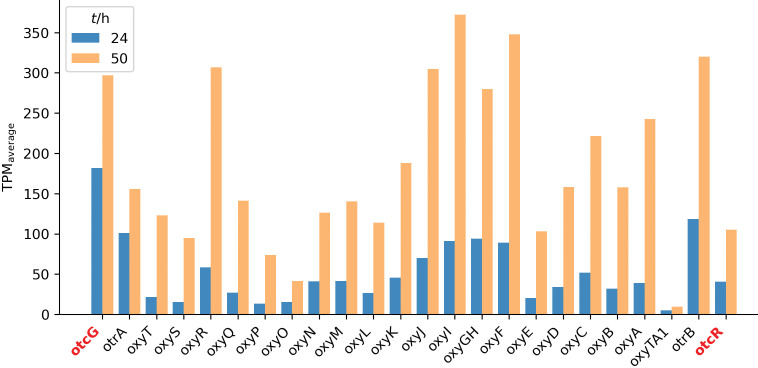
The expression (average transcripts per million (TPM) at 24 and 50 h) of each gene located in the oxytetracycline biosynthetic gene cluster in ATCC 10970

In addition to these regulators, the *otc* cluster contains the *oxyTA1* gene, which encodes a MarR-family repressor proposed to control the expression of the resistance gene *otrB*. These two genes are located immediately adjacent to each other (Fig. 6 and Fig. S1).

To further elucidate the *otc* BGC regulation in ATCC 10970, we analysed the transcriptome raw data generated by Pšeničnik *et al*. (*12*) and focused specifically on the expression profile of each gene in the *otc* BGC after 24 and 50 h (Fig. 6).

Not surprisingly, gene expression increased significantly after 50 h of incubation, which correlates well with the significant increase in OTC concentration (see raw data in (*12*)). Interestingly, only *oxyTA1* expression did not increase significantly after 50 h. This is not surprising, considering that the product of *oxyTA1* is a putative MarR family repressor protein that negatively regulates the expression of the tetracycline efflux pump gene *otrB*. The *oxyTA1* gene is divergently transcribed from *otrB*, which encodes a membrane protein responsible for OTC efflux, thereby contributing to antibiotic resistance. Thus, oxyTA1 acts as a repressor by controlling the expression of the OTC efflux pump and modulates resistance in *S. rimosus*. Therefore, it is advantageous for *S. rimosus* to promote efflux pump activity during intensive OTC biosynthesis in the cell.

#### Distantly situated regulatory elements and their role in OTC biosynthesis

**Fig. 7:**
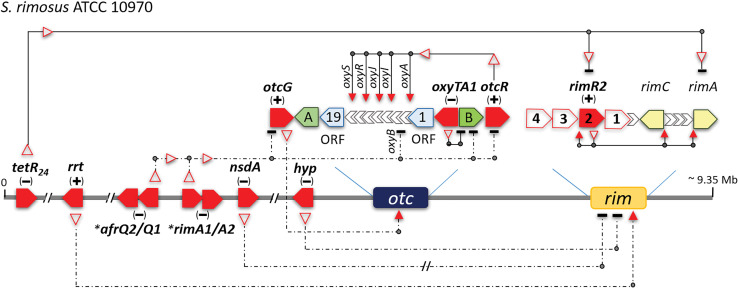
Schematic representation of regulatory genes and the antibiotic biosynthetic clusters for oxytetracycline (*otc*) and rimocidin (*rim*) in ATCC 10970. Genes encoding transcriptional activators and repressors are labelled with a plus (+) and minus (−) sign, respectively. The resistance genes *otrA* and *otrB* are denoted with solid green pentagons (labelled A and B, respectively). Experimentally characterized regulatory genes are denoted with solid red pentagons and corresponding gene names. Their protein products are denoted with red empty triangles pointing towards target genes within the clusters or towards the entire cluster when specific targets are unknown. The following is also shown: direct promoter regulation (solid lines), cascade pathway (dashed line with a double slash), pathways for which the involvement of intermediate regulators is unclear (dashed lines), activation (red bold triangles), and repression (inverted ’T’ symbols). The asterisk marks two regulatory elements (two-component systems, TCSs) that control the expression of the same genes within the OTC cluster

Beyond pathway-specific regulation, experimentally investigated regulatory elements located distantly from the OTC cluster have also been shown to modulate its expression. Bioinformatic analysis has identified 53 typical two-component systems (TCS) in *S. rimosus*, of which two were selected for experimental characterization based on their high similarity to the antibiotic-positive regulators AfsQ1/Q2 and RapA1/A2 from *S. coelicolor.* Both TCS were studied in *S. rimosus* 4018, a derivative of the parental strain 10970. The first system, designated AfrQ1Q2 (*44*), showed the highest similarity to the AfsQ1/Q2 system of *S. coelicolor*. Disruption of the response regulator *afrQ1* significantly upregulated the transcription of five genes: *oxyB* (involved in OTC biosynthesis), *otrB* and the extra-cluster *otrC* (resistance genes), and the cluster-situated regulatory genes *otcG* and *otcR* (Fig. 7). These effects were specifically observed during cultivation in minimal medium with glycine as the sole nitrogen source.

Similarly, the second system, RimA1/A2, which shares high similarity with the TCS RapA1/A2, was identified as a negative regulator of OTC production under the same nutritional conditions (*44*, *45*). Disruption of *rimA1* resulted in an identical phenotype of increased yellow-pigmented OTC production and a similar transcriptional profile to the *afrQ1* mutant. Notably, the only significant difference in the transcriptional analysis was that the *rimA1* deletion led to a several-fold higher upregulation of the regulatory gene *otcG* compared to the *afrQ1* mutant.

Altogether, the reported data show that both studied TCS act as negative regulators of OTC biosynthesis in *S. rimosus* (Fig. 7). Their overlapping phenotypes and transcriptional targets suggest they may converge on a common global regulatory node to modulate OTC biosynthesis in response to nitrogen availability.

Control of antibiotic biosynthesis by phosphate *via* the two-component PhoR/PhoP system has been well studied in several *Streptomyces* species (*46*, *47*). Early physiological studies showed that *S. rimosus* produces OTC abundantly when mycelial growth is limited by phosphate depletion. A pivotal study by McDowall *et al*. (*48*) established that this phosphate-dependent regulation occurs at the transcriptional level. They demonstrated that phosphate limitation triggers the increased expression of genes within the cluster, specifically the reductase gene *oxyR* (formerly *otcX1*) and the monooxygenase *oxyS* (formerly *otcC*). Furthermore, evidence suggests that the resistance gene *otrA* is co-transcribed with *oxyS* as part of a polycistronic message, providing a coordinated mechanism that ensures OTC resistance increases in proportion to antibiotic production. While these early studies established a transcriptional link to phosphate availability, the specific role of the PhoR/PhoP system in *S. rimosus* has yet to be experimentally validated, as no studies involving the genetic manipulation of this system have been reported to date.

#### Cluster-situated and distal regulatory elements that govern RIM biosynthesis

Although ATCC 10970 produces both OTC and RIM under laboratory conditions, genes involved in the regulation of RIM synthesis have mainly been studied in *S. rimosus* M527. Interestingly, although this strain carries a BGC for OTC production that is highly similar (>90 %) to that of ATCC 10970, it does not produce OTC under the tested laboratory conditions (*49*). The *S. rimosus* M527 strain was classified as *S. rimosus* based on 16S rRNA gene sequence analysis. Thus, we first compared the *S. rimosus* M527 genome with that of ATCC 10970 (*50*) to assess the conservation of genes involved in *rim* BGC regulation.

**Fig. 8:**
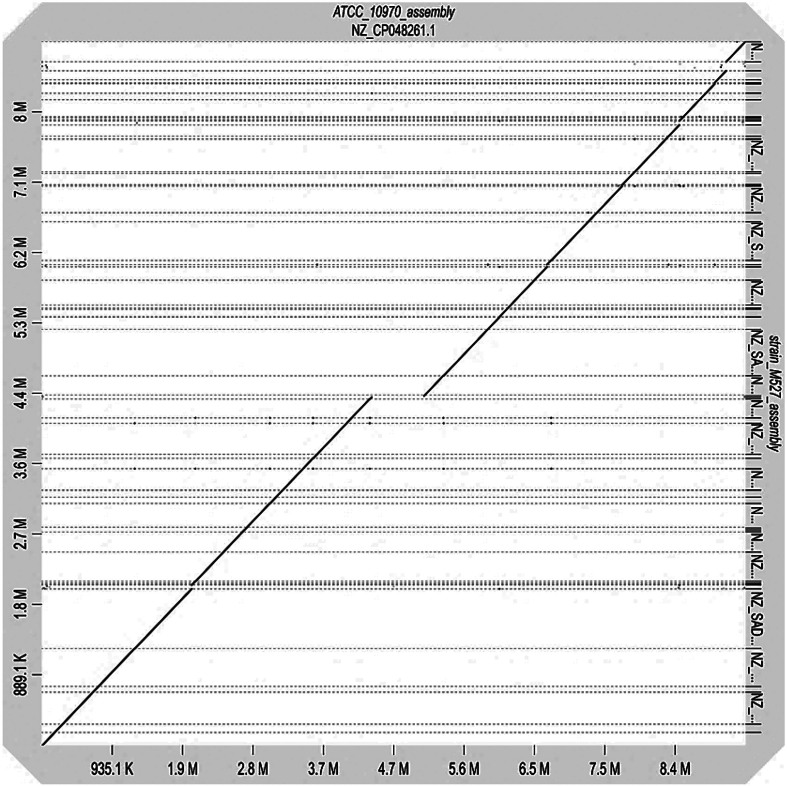
Whole-genome dot plot alignment of ATCC 10970 (X-axis, NZ_CP048261.1) and *S. rimosus* M527 (Y-axis, strain_M527_assembly). The plot illustrates overall genomic synteny and identifies regions of conservation (diagonal lines) and structural variations

A whole-genome alignment between ATCC 10970 (assembly NZ_CP048261.1; *23*) and *S. rimosus* M527 was performed using D-Genies (*51*) (Fig. 8). The dot plot visualization of this comparison (Fig. 8) reveals a high degree of collinearity between the two genomes, indicated by the strong diagonal line. This suggests large syntenic blocks and a conserved gene order across most chromosomes.

Some minor breaks in the main diagonal and smaller off-diagonal alignments are visible, indicating potential genomic rearrangements such as inversions, translocations, or insertions/deletions (indels) that differentiate the two strains. The alignment statistics (Fig. 8) show that 92.12 % of the queried genome (M527 against ATCC 10970, as reference) aligns with over 75 % sequence identity. A small fraction, 7.73 %, shows no match, and 0.15 % aligns with less than 25 % identity, highlighting regions of significant divergence or unique genomic content in one of the strains. Given the high genomic similarity between the two strains, we next closely examined the presence and similarity of all experimentally characterized regulatory genes known to influence OTC and RIM production in both strains.

Analysis of the regulatory genes *otcG*, *oxyTA*1, and *otcR* in the *otc* BGC (Fig. 7) showed that they are 100 % identical in both strains. Our results indicate that the *otc* BGCs of the two *S. rimosus* strains share high overall similarity (>90 %), and OTC production was successfully activated in M527 (*49*). Thus, we propose that the lack of OTC production in wild-type *S. rimosus* M527 is most likely due to differences in the global regulation of OTC biosynthesis and may be triggered under different environmental conditions.

As mentioned earlier, ATCC 10970 and M527 strains produce RIM in cultivation medium favouring OTC production (*17*). Genome sequence analysis of *S. rimosus* M527 revealed a BGC responsible for RIM production and, based on sequence similarity, predicted four regulatory genes (*rim1* to *rim4*) located in this cluster (*52*). Phylogenetic analysis showed that Rim2, Rim3 and Rim4 share high similarity with the well-characterized LAL subfamily of LuxR transcriptional regulators, while Rim1 is more closely related to the PAS-LuxR family (Fig. 7. (*52*-*54*)). In addition, *rim2* overexpression most significantly increased antibiotic production, while deletion of this gene showed that it is essential for RIM biosynthesis. Thus, it is not surprising that its regulatory role in *rim* BGC is the best characterized so far. RIM2 specifically binds to the promoter regions of PKS *rimA*, *rimC* and *rimR2*, thereby activating the transcription of structural genes while simultaneously regulating its own expression, which exerts a positive effect on antibiotic production (*52*). All regulatory proteins in ATCC 10970 show nearly 100 % identity to their orthologues in the *S. rimosus* M527 strain; Rim1, Rim3 and Rim4 show 100 % identity, while Rim2 shows 99.13 % similarity. In addition, inspection of the promoter regions (300 bp) of the *rim1*–*rim4* genes revealed over 99.3 % sequence identity, strongly suggesting that regulation of these genes is also conserved in both strains (Fig. 7).

In addition to the regulatory genes within the *rim* BGC, several studies have reported other regulatory genes located distantly from this BGC in strain M527. Among them is the recently discovered gene encoding the transcriptional regulator TetR24 (*55*), assigned to the TetR family based on its sequence and structure. We also identified the gene encoding an identical protein in ATCC 10970 (Fig. 7). This finding is not surprising, as the members of this family regulate diverse processes in bacteria, including antibiotic biosynthesis, and are commonly found in *Streptomyces* genomes (*56*). Deletion of *tetR24* in the M527 strain and transcriptional analysis showed that TetR24 acts as a negative regulator of RIM biosynthesis. It has been reported that TetR24 binds to the *rimR2* and *rimA* promoter regions, where two binding sites with a conserved motif (GGAG/ACTCG/C) were identified (*55*). Our analysis of the *rimR2* and *rimA* promoter regions in ATCC 10970 revealed complete conservation of the TetR24 binding sites upstream of both genes, matching the previously reported sequences containing a conserved motif: TGCGTATCACCTCACGCAA**GGAACTCC** for *rimR2* and A**GGAGCTCG**TGTCCGAACCCCGG for *rimA*. Given that the *tetR24* genes in both strains encode identical TetR24 proteins and that the binding motif is conserved, it is likely that TetR24 regulates *rimR2* transcription in the same manner in both strains.

An additional gene, *nsdA*, a negative regulator of RIM antibiotic production and sporulation, has also been identified in *S. rimosus* M527 (*53*), based on its sequence similarity with NsdA from *S. coelicolor*. NsdA is conserved and widely distributed across *Streptomyces* species (*57*), suggesting an important role in regulating morphological and physiological differentiation, at least in sporulating *Streptomyces* species. Thus, it is not surprising that we identified a 100 % conserved orthologous *nsdA* gene in the genome of the reference strain ATCC 10970, when using the nsdA_sr_ sequence from strain M527 as a query.

Additionally, the 300 bp upstream region of *nsdA* was also 100 % conserved, suggesting that the transcriptional regulation of this gene is conserved between the two strains. In *S. coelicolor*, BldD binds the promoter region of *nsdA*, placing *nsdA* within the BldD regulon and identifying it as part of a complex regulatory cascade governed by this global regulator. Here, we did not identify a motif matching the BldD binding site in the upstream region of *nsdAsr*. However, the possibility that this regulatory cascade is conserved in *S. rimosus* cannot be excluded, as not all known BldD-regulated genes contain recognizable BldD motifs in their promoter regions (*58*). Disruption of the *nsdA* gene in *S. rimosus* M527 increases antibiotic production and accelerates sporulation (*53*), consistent with previous findings in other *Streptomyces* species, including *S. coelicolor*, *S. lividans* and *S. bingchenggensis* (*59*). Recent transcriptomic and ChIP-seq analyses have provided new insights into the regulatory mechanisms controlled by NsdA_sr_. This repressor downregulates metabolic pathways involved in butyryl-CoA and malonyl-CoA biosynthesis, precursors of rimocidin biosynthesis, as well as global protein synthesis, thereby modulating antibiotic production (*54*). Given the ubiquity of this regulator in streptomycetes, all these findings indicate that *nsdA* is a promising target for genetic manipulation in industrial strain improvement programs (*57*).

Finally, two additional genes, *rrt* and *hyp*, involved in the regulation of the *rim* operon in *S. rimosus* M527 (*60*), have recently been reported. Both genes are located outside the *rim* BGC. The first encodes the Rrt protein, which functions as a positive regulator of RIM production. Notably, identical or highly similar Rrt protein sequences have been identified in several *Streptomyces* strains, including *S. rimosus* R6-500 and WT5260 (also designated as ATCC 10970) (*60*). Therefore, our discovery of a gene encoding a 100 % identical Rrt protein in the reference strain ATCC 10970 (Fig. 7) was not surprising, suggesting a conserved biological role for this protein in the regulation of antibiotic production in many *Streptomyces* strains. In contrast to this positive regulator, the Hyp (from hypothetical) protein negatively regulates RIM synthesis (*60*). A 100 % identical Hyp protein, encoded outside the *rim* BGC (Fig. 7), is also present in the reference strain ATCC 10970. This conservation suggests that it plays the same regulatory role in repressing RIM biosynthesis in both *S. rimosus* strains, ATCC 10970 and M527. Interestingly, transcriptional analyses of RIM biosynthetic genes (*rimA, rimB, rimE, rimG*) and regulatory genes (*rim1*, *rim2*) revealed coordinated expression changes, with decreases in the absence of the Rrt activator and increases in the absence of the Hyp repressor, respectively (*60*). This conserved trend in transcriptional profiles suggests that Rrt and Hyp either regulate the same cluster regulators (*rim1* and *rim2*) or act through a shared signalling pathway involving an as-yet-undiscovered intermediate regulator. In conclusion, analyses of all regulatory elements described in the literature (Fig. 7) do not suggest ‘cross-regulation’ between OTC and RIM BGCs. This may be the main reason why the inactivation of the *rim* BGC does not affect OTC biosynthesis.

## CONCLUSIONS

Based on bioinformatic analyses, we have clearly demonstrated that most biosynthetic gene clusters (BGCs) identified in *Streptomyces rimosus* ATCC 10970, which encode the biosynthesis of secondary metabolites, are weakly expressed or entirely silent. We have shown that for the major metabolites RIM and OTC, which both require the same building block (malonyl-CoA) for their biosynthesis, substrate supply is not a bottleneck under the applied cultivation conditions. Additionally, in agreement with published data, this result suggests that no significant inter-regulation exists between these two BGCs.

In contrast, the inactivation of BGC 42, which encodes an as yet uncharacterised metabolite and is located at the other end of the ATCC 10970 chromosome, far from *otc* BGC 9, has significant positive effects on OTC production. This result demonstrates that these two BGCs encode competitive pathways. However, as BGC 42 is entirely transcriptionally silent, this result also suggests the existence of unknown regulatory system(s).

By deleting otherwise silent BGCs, we may have affected other aspects of gene regulation, as a consequence of the changes in chromosome topology. The influence of chromosome topology may be much greater than previously thought, and we believe that this is a topic that we should investigate further. Engineered *Streptomyces rimosus* host strains thus represent a very good model system to study the expression of ’silent’ biosynthetic gene clusters.

## SUPPLEMENTARY MATERIAL



**Table S1:** Bacterial strains used in this study

Strain	Description	Source
*E*. *coli* DH10β	F-*end*A1 *rec*A1 *gal*E15 *gal*K16 *up*G *rps*LΔ*lac*X74 Φ80*lac*ZΔM15 *ara*D139 Δ(*ara*,*leu*)7697 *mcr*A Δ(*mrr*-*hsd*RMS-*mcr*BC) λ-; used in plasmid cloning and construction steps	Invitrogen, Carlsbad, USA
*E*. *coli* ET12567/pUB307	F-*dam*13::Tn9 *dcm*6 *hsd*M *hsd*R *rec*F143 *zjj*-202::Tn*10* *gal*K2 *gal*T22 *ara*14 l*ac*Y1 *xyl*5, *leu*B6, *thi*1, *ton*A31 *rpsL*136, *his*G4, *tsx*78, *mtl*I *glnV*44, plasmid pUB307; preparation of non-methylated DNA for conjugation	(*13*)
*S. rimosus* ATCC 10970	OTC producer, wild type	(*14*)
*S. rimosus* ATCC 10970 Δotc	Strain with OTC BGC deletion	(*15*)
*S. rimosus* ATCC 10970 Δotc Δ145kb *S. rimosus* ATCC 10970 ΔBGC42	Strain with OTC BGC deletion and 145 kb deletion Strain with BGC 42 deletion	(*12*) This work

BGC=biosynthetic gene cluster, OTC=oxytetracycline

**Table S2:** Plasmids used in this study

Plasmid	*N*/kbp	Key features	Source
pAB04	7.1	ΦC31, Apr^R^, Tio^R^, promotor erm*	(*16*)
pREP_P1_Cas9	10.8	pIJ101 replicon, Apr^R^	(*12*)
pGH	2.9	Amp^R^, plasmid on which we obtained synthesized DNA fragments	ATG:Biosynthetics GmbH, Merzhausen, Germany
pVF	8.9	pIJ101 replicon, Amp^R^	(*16*)
pAB13	8.1	Derived from pKC1139, Apr^R^, Erm^R^, with a thermo-sensitive replicon	(*15*)
pREP_GBG42_1a1b pREP_GBG42_2a2b pREP_GBG42_3		Plasmid pREP_P1_Cas9_tio, containing homologous regions and guide RNAs (gRNA 1a and 1b) for deletion of BGC 42 Plasmid pREP_P1_Cas9_tio, containing homologous regions and guide RNAs (gRNA 2a and 2b) for deletion of BGC 42 Plasmid pREP_P1_Cas9_tio, containing homologous regions and guide RNAs (gRNA 3) for deletion of BGC 42	This work This work This work

BGC=biosynthetic gene cluster

**Table S3:** Putative biosynthetic gene clusters (BGCs) identified in the *Streptomyces rimosus* ATCC 10970 genome based on antiSMASH 6.0 analysis (*22*) and isolated metabolites

Cluster no. in ATCC 10970	Type of BGC*	Position**	Most similar known BGC (Similarity/%)	Metabolites detected in culture extract in our study
Chromosome 1	NRPS fragment	90930-97183	Paromomycin (7)	Guanipiperazines A and B
2	PKS type I-NRPS	188819-209069	NA	
3	Terpene	209478-217564	Isorenieratene (85)	
4	NRPS	225846-253508	Atratumycin (13)	
5	PKS type I	321687-347936	Sceliphrolactam (32)	
6	PKS type I	399364-499930	Nystatin A1 (72)	Rimocidin, CE108, amide, CE108
7	NRPS	513458-544839	Qinichelins (22)	
8	Lassopeptide/RiPP	579166-586929	Lagmysin (80)	
9	PKS type II	628015-655782	Oxytetracycline (100)	Oxytetracycline
10	PKS type I	786388-806568	NA	
11	Lantipeptide/RiPP	899955-907971	NA	
12	PKS type I	922668-952762	Spiroindimicins/Indimicins/lynamicins (6)	
13	NRPS-like	989591-1015728	Stenothricin (13)	
14	NRPS-PKS type	1034416-1064312	Rimosamide (92)	Rimosamides A–D
15	NRPS	1095198-1140552	Daptomycin (14)	
16	Arylpolyene	1162316-1218483	Herboxidiene (3)	
17	Terpene	1386125-1399202	Hopene (76)	
18	NRPS	1568818-1619165	Isocomplestatin (93)	
19	Melanin	1756702-1763509	Bagremycin A/B (11)	
20	Lantipeptide/RiPP	2189994-2200974	NA	
21	NRPS	2267432-2288427	Streptobactin (70)	Streptobactin
22	NRPS	2320795-2393710	Ulleungmycin (36)	Longicatenamycin
23	NRPS-PKS type	3089234-3116494	Tyrobetaine (100)	Tyrobetaine, tyrobetaine-2, chlorotyrobetaine, chlorotyrobetaine-2
24	NRPS	4147387-4194710	Mannopeptimycin (22)	
25	Arylpolyene	4258214-4287270	Fusaricidin B (25)	
26	NRPS	4793268-4840550	Ishigamide (61)	
27	Lassopeptide/RiPP	5834963-5841023	Moomysin (50)	
28	Lantipeptide/RiPP	6587454-6598475	SAL-2242 (77)	
29	Terpene	6817266-6819473	Geosmin (100)	
30	Ectoine	7244554-7247941	Ectoine (100)	Ectoine
31	Siderophore	7331013-7336394	Desferrioxamine E (100)	Deferoxamin
32	Siderophore	7433301-7442083	NA	
33	Terpene	8052420-8062100	NA	
34	PKS type I-NRPS	8343488-8380063	Marinacarboline (23)	
35	NRPS	8502626-8519135	Deimino-antipain (66)	Chymostatin A, B, C
36	NRPS-like	8619558-8643234	NA	
37	PKS type I or PKS type I saccharide	8655191-8687260	Tetronasin (9)	
38	NRPS	8692521-8715452	Mannopeptimycin (14)	
39	Terpene	8720327-8725815	NA	
40	Other NRPS-like	8825293-8867032	A83543A (8)	
41	Butyrolactone	8884982-8896849	Cyphomycin (11)	
42	PKS type I-NRPS	8971199-8996615	NA	
43	NRPS	9016185-9065343	Teicoplanin (28)	
44	Nucleoside	9075785-9088816	Pseudouridimycin (68)	Pseudouridimycin
45	NRPS	9091105-9149322	NA	Momomycin
46	NRPS	9257979-9275999	NA	
Plasmids				
1 P	PKS type I	143989-163050	Kanamycin (1)	
2 P	NRPS	215829-230795	NA	

*Refers to the type of biosynthetic enzyme complex involved in the formation of putative secondary metabolites. The BGCs in this table correspond to the designation numbers in Fig. 1 and Fig. 2. **The location of the identified BGCs corresponds to the genome sequence of GenBank assembly accession no. GCF_006229535.1 (*17*). NA=not applicable, NRPS=non-ribosomal peptide synthetase, PKS=polyketide synthase, RiPP=ribosomally synthesized and post-translationally modified peptides

Data S1. High-resolution mass spectrometry (HRMS) spectrum exhibited a protonated molecular ion peak at *m/z*=529.3830 corresponding to [M+H]^+^ for the molecular formula C_40_H_49_. This value is in excellent agreement with the calculated exact mass of 529.38288, thereby validating the proposed molecular composition.
