# Supplementary material for: Modulating Complex Secondary Metabolism in Streptomyces rimosus by Targeted Genome Engineering
Source: Food Technol Biotechnol. 2026 Feb 15;64(1):97–112. doi: 10.17113/ftb.64.01.26.9441 (PMC13098539; doi:10.17113/ftb.64.01.26.9441)
Supplement: Supplementary file 1 [file FTB-64-97-S1.pdf]

Back to article

Table S1. Bacterial strains used in this study

Strain	Description	Source
E. coli DH10β	F-*end*A1 *rec*A1 *gal*E15 *gal*K16 *upG rps*LΔ*lac*X74 Φ80*lac*ZΔM15 *ara*D139 Δ(*ara,leu*)7697 *mcr*A Δ(*mrr-hsd*RMS-*mcr*BC) λ-; used in plasmid cloning and construction steps	Invitrogen, Carlsbad, USA
E. coli ET12567/pUB307	F-*dam*13::Tn9 *dcm*6 *hsdM hsdR recF*143 *zjj*-202::Tn*10* *gal*K2 *gal*T22 *ara*14 lacY1 *xyl*5, *leu*B6, *thi*1, *ton*A31 *rpsL*136, *his*G4, *tsx*78, *mtl*I *glnV*44, plasmid pUB307; preparation of non-methylated DNA for conjugation	[13]
S. rimosus ATCC 10970	OTC producer, wild type	[14]
S. rimosus ATCC 10970 Δotc	Strain with OTC BGC deletion	[15]
S. rimosus ATCC 10970 Δotc Δ145kb	Strain with OTC BGC deletion and 145 kb deletion	[12]
S. rimosus ATCC 10970 ΔBGC42	Strain with BGC 42 deletion	This work

BGC=biosynthetic gene cluster, OTC=oxytetracycline

Back to article

Table S2. Plasmids used in this study

Plasmid	*N*/kbp	Key features	Source
pAB04	7.1	ΦC31, Apr^R, Tio^R, promotor *erm**	[16]
pREP_P1_Cas9	10.8	pIJ101 replicon, Apr^R	[12]
pGH	2.9	Amp^R, plasmid on which we obtained synthesized DNA fragments	ATG:Biosynthetics GmbH, Merzhausen, Germany
pVF	8.9	pIJ101 replicon, Amp^R	[16]
pAB13	8.1	Derived from pKC1139, Apr^R, Erm^R, with a thermo-sensitive replicon	[15]
pREP_GBG42_1a1b		Plasmid pREP_P1_Cas9_tio, containing homologous regions and guide RNAs (gRNA 1a and 1b) for deletion of BGC 42	This work
pREP_GBG42_2a2b		Plasmid pREP_P1_Cas9_tio, containing homologous regions and guide RNAs (gRNA 2a and 2b) for deletion of BGC 42	This work
pREP_GBG42_3		Plasmid pREP_P1_Cas9_tio, containing homologous regions and guide RNAs (gRNA 3) for deletion of BGC 42	This work

BGC=biosynthetic gene cluster

Back to article

Table S3. Putative biosynthetic gene clusters (BGCs) identified in the *Streptomyces rimosus* ATCC 10970 genome based on antiSMASH 6.0 analysis [22] and isolated metabolites

Cluster no. in ATCC 10970	Type of BGC*	Position**	Most similar known BGC (Similarity/%)	Metabolites detected in culture extract in our study
Chromosome				
1	NRPS fragment	90930–97183	Paromomycin (7)	Guanipiperazines A and B
2	PKS type I-NRPS	188819–209069	NA	
3	Terpene	209478–217564	Isorenieratene (85)	
4	NRPS	225846–253508	Atratumycin (13)	
5	PKS type I	321687–347936	Sceliphrolactam (32)	
6	PKS type I	399364–499930	Nystatin A1 (72)	Rimocidin, CE108, amide, CE108
7	NRPS	513458–544839	Qinichelins (22)	
8	Lassopeptide/RiPP	579166–586929	Lagmysin (80)	
9	PKS type II	628015–655782	Oxytetracycline (100)	Oxytetracycline
10	PKS type I	786388–806568	NA	
11	Lantipeptide/RiPP	899955–907971	NA	
12	PKS type I	922668–952762	Spiroindimicins/ Indimicins/ lynamicins (6)	
13	NRPS-like	989591–1015728	Stenothricin (13)	

Table S3. continued

Cluster no. in ATCC 10970	Type of BGC*	Position**	Most similar known BGC (Similarity/%)	Metabolites detected in culture extract in our study
14	NRPS-PKS type	1034416–1064312	Rimosamide (92)	Rimosamides A–D
15	NRPS	1095198–1140552	Daptomycin (14)	
16	Arylpolyene	1162316–1218483	Herboxidiene (3)	
17	Terpene	1386125–1399202	Hopene (76)	
18	NRPS	1568818–1619165	Isocomplestatin (93)	
19	Melanin	1756702–1763509	Bagremycin A/B (11)	
20	Lantipeptide/RiPP	2189994–2200974	NA	
21	NRPS	2267432–2288427	Streptobactin (70)	Streptobactin
22	NRPS	2320795–2393710	Ulleungmycin (36)	Longicatenamycin
23	NRPS-PKS type	3089234–3116494	Tyrobetaine (100)	Tyrobetaine, tyrobetaine-2, chlorotyrobetaine, chlorotyrobetaine-2
24	NRPS	4147387–4194710	Mannopeptimycin (22)	
25	Arylpolyene	4258214–4287270	Fusaricidin B (25)	
26	NRPS	4793268–4840550	Ishigamide (61)	
27	Lassopeptide/RiPP	5834963–5841023	Moomysin (50)	
28	Lantipeptide/RiPP	6587454–6598475	SAL-2242 (77)	
29	Terpene	6817266–6819473	Geosmin (100)	
30	Ectoine	7244554–7247941	Ectoine (100)	Ectoine
31	Siderophore	7331013–7336394	Desferrioxamine E (100)	Deferoxamin
32	Siderophore	7433301–7442083	NA	
33	Terpene	8052420–8062100	NA	
34	PKS type I-NRPS	8343488–8380063	Marinacarboline (23)	
35	NRPS	8502626–8519135	Deimino-antipain (66)	Chymostatin A, B, C
36	NRPS-like	8619558–8643234	NA	
37	PKS type I or PKS type I saccharide	8655191–8687260	Tetronasin (9)	
38	NRPS	8692521–8715452	Mannopeptimycin (14)	
39	Terpene	8720327–8725815	NA	
40	Other NRPS-like	8825293–8867032	A83543A (8)	
41	Butyrolactone	8884982–8896849	Cyphomycin (11)	
42	PKS type I-NRPS	8971199–8996615	NA	
43	NRPS	9016185–9065343	Teicoplanin (28)	
44	Nucleoside	9075785–9088816	Pseudouridimycin (68)	Pseudouridimycin
45	NRPS	9091105–9149322	NA	Momomycin
46	NRPS	9257979–9275999	NA	
Plasmids				
1 P	PKS type I	143989–163050	Kanamycin (1)	
2 P	NRPS	215829–230795	NA	

*Refers to the type of biosynthetic enzyme complex involved in the formation of putative secondary metabolites. The BGCs in this table correspond to the designation numbers in **Fig. 1** and **Fig. 2**. **The location of the identified BGCs corresponds to the genome sequence of GenBank assembly accession no. GCF_006229535.1 [*17*]. NA=not applicable, NRPS=non-ribosomal peptide synthetase, PKS=polyketide synthase, RiPP=ribosomally synthesized and post-translationally modified peptides

Data S1. High-resolution mass spectrometry (HRMS) spectrum exhibited a protonated molecular ion peak at *m/z*=529.3830 corresponding to $[M+H]^+$ for the molecular formula $C_{40}H_{49}$. This value is in excellent agreement with the calculated exact mass of 529.38288, thereby validating the proposed molecular composition.

Back to article

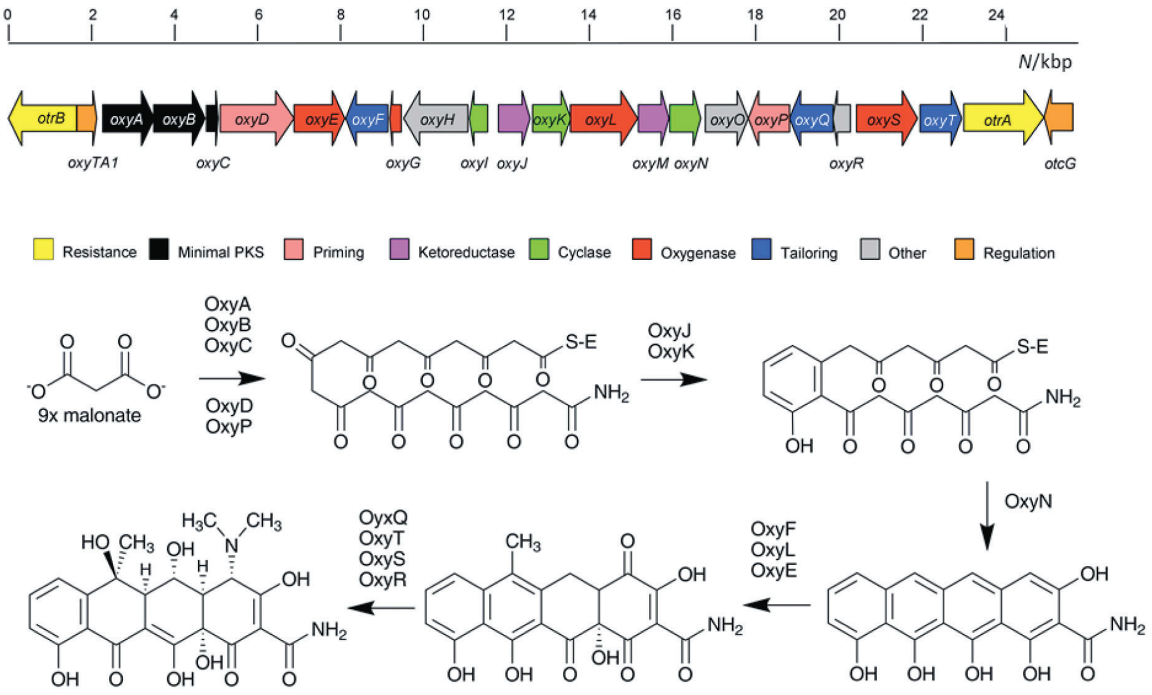

Fig. S1. Proposed biosynthetic pathway and genes involved in oxytetracycline (OTC) biosynthesis. PKS=polyketide synthase
